# Evaluating the cost-effectiveness of preventive zinc supplementation

**DOI:** 10.1186/1471-2458-14-852

**Published:** 2014-08-15

**Authors:** Günther Fink, Jesse Heitner

**Affiliations:** Department of Global Health and Population, Harvard School of Public Health, 665 Huntington Avenue, 02115 Boston, MA USA

**Keywords:** Zinc supplementation, Cost-effectiveness, Diarrhea, ALRI

## Abstract

**Background:**

Even though the WHO currently recommends zinc for diarrhea management, no consensus has been reached with respect to routine distribution of zinc for preventive reasons. We reviewed the health impact of preventive zinc interventions, and evaluated the relative cost effectiveness of currently feasible interventions.

**Methods:**

Using the latest relative risk estimates reported in the literature, we parameterized a health impact model, and calculated the expected benefits of zinc supplementation in a representative low-income country. We then computed the cost and cost-effectiveness for three delivery mechanisms: the direct distribution of zinc supplements, the distribution of micronutrient biscuits including zinc, and the distribution of zinc through water filtration systems.

**Results:**

Combining all health outcomes and impact estimates, we find that systematic zinc supplementation among children of ages one to five would avert 1.423 DALYs per 100 households and year in least developed countries. The estimated cost per DALY is US$ 606 for pill supplementation, US$ 1211 for micronutrient biscuits, and US$ 879 per DALY saved for water filtration systems.

**Conclusions:**

Preventive zinc supplementation to children of ages 1–5 appears to be a highly cost-effective intervention in typical developing country settings. More research will be needed to determine the most effective mechanism to deliver zinc to this target population.

## Background

Zinc affects the human body through a large number of channels affecting not only cell division, protein synthesis and growth, but also gene expression and a variety of reproductive and immunologic functions [1]. The absence of sufficient levels of zinc in the human body is associated with a large number of adverse health outcomes, including reduced physical growth, lower immune competence and suppressed neural development [2]. The global burden of disease due to zinc deficiency is estimated at 800,000 excess deaths among children under 5 years of age annually, most of which are attributed to pneumonia, diarrhea and malaria [3]. Even though the World Health Organization (WHO) recommends zinc for diarrhea management [4, 5], no consensus has been reached with respect to routine distribution of zinc for preventive reasons.

A large and rapidly growing literature has explored the degree to which zinc supplementation can improve population health in general, and child health in particular [6–10]. While some studies have documented negative effects of zinc supplementation on iron absorption [11], the adverse effects of zinc supplementation on concentrations of hemoglobin, serum ferritin, and serum copper appear limited [12–15]. Given this, the case for more comprehensive zinc supplementation programs seems promising, and naturally raises the question regarding the potential delivery mechanisms for zinc and their relative cost effectiveness.

While direct dietary supplementation is the most commonly used approach in trials, several other delivery mechanisms have been proposed in the literature: Hess and Brown [16] highlight the potentially large benefits of food fortification with zinc oxide or zinc sulfate; Gibson and Anderson [17] review the potential benefits of dietary diversification, and Hotz [7] makes a case for a more general biofortification of staple crops in developing countries. Even though the WHO currently considers both zinc *supplementation* and zinc *fortification* as potentially cost-effective interventions [18], a general delivery of zinc through centrally produced wheat or other staple foods may be difficult in many developing countries today due the small scale and decentralized structure of most agricultural sectors in developing countries, and because fortification programs may miss rural populations and those urban poor who consume few processed foods [19]. While bio-fortification appears to be an attractive and culturally appropriate alternative in principal [19, 20], the upfront cost of genetically modified crop development appears high [19], so that implementation at a meaningful scale is highly unlikely to happen in the near future. In this paper, we focus on mechanisms to implement large scale zinc supplementation which appear feasible in the near term, compute their relative cost-effectiveness and discuss both the feasibility and scalability of each mechanism.

## Methods

In order to assess the cost-effectiveness of zinc supplementation under the various distribution scenarios, we proceeded in four steps. In a first step, a systematic literature review was conducted using both the Google Scholar and PubMed search engines. Given the large number of review studies in the area, the literature review was restricted to meta-studies based on randomized controlled trials with a focus on preventive zinc supplementation. All review studies meeting these criteria were independently evaluated by both authors. Mismatching coding was reconciled by a joint review of the relevant papers.

In a second step, the meta-studies were used to parameterize a health impact model to calculate the expected benefits of zinc supplementation in an average low-income country. The model was based on the standard cost-effectiveness procedures outlined by the Panel on Cost Effectiveness in Health and Medicine [21], taking the perspective of an external financing agency. Following WHO guidelines [22], health benefits were calculated in terms of disability adjusted life years (DALYs) saved through one year of supplementation for groups of 100 households. Following Stein [19], DALYs were computed using the following equation:
1

where *T*_*j*_ is the number of people in target group *j*, *M*_*j*_ is the mortality rate associated with the condition in target group *j*, *L*_*ij*_ is the average remaining life expectancy for target group *j*, *I*_*ij*_*is the* incidence rate of health outcome *i* in group *j D* is the corresponding disability weight, *d* is the duration of the health outcome, and *r* is the temporal discount rate, which we assume to be 3% as recommended [22]. Disability weights for both were taken from standard Global Burden of Disease Database (GBD) [23]. Morbidity effects were assumed to be mutually exclusive, so that each case of child morbidity was either classified as diarrhea, malaria or acute lower respiratory illness (ALRI) only. Mortality effects were modeled as potential outcome (consequence) of child morbidity.

In order to calibrate the model to a representative developing country setting, data on average household characteristics in countries classified as *least developed* were extracted from the United Nations World Population Prospects data base [24]. Seventy-seven countries were classified as “least *developed*”, with a total population of about 900 Million people across 3 continents. Incidence data for both diarrhea and ALRI in developing countries was derived from representative household surveys data collected through the Demographic and Health Surveys (DHS) [25]. These surveys contain two-week-disease-specific prevalence data, which were converted to annual incidence numbers. Baseline data on child mortality was downloaded from UNICEF’s State of The World’s Children database [26]. Applying Equation 1 to the data from UNICEF, the GBD, and the DHS surveys, number of DALYs currently being lost for a synthetic set of 100 households in “least developed” countries was calculated.

In a third step, we analyzed the relative effectiveness of three potential delivery mechanisms for zinc. The most commonly studied delivery mechanism for zinc supplementation in clinical trials is direct distribution of zinc pills [27–29]. An alternative approach to zinc supplementation is the distribution of micronutrient biscuits; such biscuits (or sprinkles for cereals) have been shown to be highly effective in reducing anemia and increasing plasma zinc among school children [30]. The third delivery mechanisms considered was the deployment of home-based water filtration systems. Such systems are currently in the trial stage, and designed to directly dispense zinc into drinking water as part of the water filtration process.

Relative risks of diarrhea and ALRI for interventions supplying zinc were taken from published literature on zinc supplementation and applied to current incidence rates for these diseases in the synthetic set of 100 representative households. Using these new incidence rates, the DALYs that would still be lost amongst the synthetic households was calculated, and the difference from the DALYs in step two was taken as the incremental reduction in DALYs from providing these interventions to the 100 synthetic households.

In the fourth step, cost estimates for each of the delivery mechanisms analyzed were compiled from recently published trials as well as other online resources. For all interventions, the most recent cost estimates were taken. Given that no evidence was found suggesting an increase in the absolute prices of either delivery mechanism, no inflation adjustments were made. Dividing the estimated cost of delivery by the incremental reduction in DALYS from providing each intervention yielded the incremental cost effectiveness of providing each intervention to 100 synthetic households given current disease trends. For all cost-effectiveness calculations the assumed counterfactual was no intervention at all, i.e. that none of the other interventions considered would be implemented, which means that incremental cost-effectiveness estimates equal mean cost-effectiveness estimates.

All data utilized in the analysis came from existing sources, and no human subjects research was performed.

## Results

### Literature review

The literature search revealed a remarkably large number of studies investigating the effects of zinc. More than two million studies contained zinc as keywords in Google Scholar, and more than 20,000 studies were linked to zinc supplementation, prevention and mortality in the same search engine. From the 199 meta or review studies found in Google Scholar, 186 were unrelated to morbidity or mortality prevention or were merely citations, corrections, posters, or used otherwise inappropriate formats, 4 were unrelated to dietary intake in humans, and 3 contained general discussions rather than empirical evidence. This left six articles from the Google Scholar search which contained meta analyses of the relationship between zinc supplementation and mortality or morbidity prevention. A second search on meta-studies related to zinc was conducted in PubMed. Based on the keyword “zinc” and limited to meta-analysis, 103 studies focusing on human subjects were identified. 35 were excluded because they were not related to zinc supplementation; 25 were related to treatment rather than to prevention, 14 measured risk factors (such as height or weight or chemical concentrations) rather than health outcomes, 5 studies were not based on clinical trials; and one study was excluded due to the lack of a comparable placebo group. Six studies were older versions of updated articles already included and thus also omitted, which resulted in a final selection of 17 meta-analysis from the PubMed search, four of which were also found in the Google Scholar search. Finally, two articles were included from other previously known sources and searches. This made a total of 21 studies selected for the final review.

### Health impact of zinc supplementation

#### Diarrheal diseases

Aggarwal et al. [31] reviewed all controlled trials on diarrheal outcomes published up to November 2005. The pooled analysis yielded a statistically significant relative risk (RR) for mild or severe diarrhea of 0.86, with a 95% confidence interval of [0.79-0.95]. The RR for severe diarrhea was 0.85 [0.75-0.95], and 0.75 [0.57-0.98] when looking at persistent diarrhea. Brown et al. [13] included 87 articles published before May 2007. The pooled analysis found a significant protective effect: RR = 0.80, 95% CI [0.71-0.90]. No effects were found in studies with mean age of children 12 months or younger. The effect was largest for children 12 months or older RR = 0.73, 95% CI [0.61 – 0.87]. Yakoob et al. [9] reviewed 14 studies qualified as “high quality”, and found a pooled risk reduction of 13% (RR = 0.87; 95% CI [0.81, 0.94]). Patel et al. [32] conducted the most comprehensive review of the literature on diarrheal diseases to date, reviewing all studies up to 2011 including three preceding meta-studies. The pooled analysis yielded a summary rate ratio of 0.91 [0.87-0.95]. The estimated prevalence effect was larger, with a risk ratio of 0.81 [0.75-0.88]. When adjusting for cluster randomization, the estimated range was [0.88-0.94].

#### Respiratory infections

Aggarwal et al. [31] reviewed 12 studies investigating the effect of zinc supplementation on respiratory illness. The summary relative risk was 0.92 [0.85-0.99]. Four reviewed studies investigated acute lower respiratory infection (ALRI), with a pooled RR of 0.80 [0.70-0.92]. Brown et al. [8] reviewed 16 comparisons based on 12 studies on ALRI, and found a pooled relative ALRI risk of 0.85 [0.75-0.97]. With a stringent definition (physician exam or counting respiratory rates, 9 comparison groups) a relative risk of 0.79 [0.67-0.94] was found. Roth et al. [28] updated the previous reviews with a particular focus on ALRI. A total of 10 studies were included. Using the least specific definition provided to address differences in ALRI diagnosis, the pooled estimate was an insignificant rate ratio of 0.94 [0.88-1.01]. Using the most specific definition provided by each paper, the pooled estimate was an insignificant protective rate ratio of 0.86 [0.74-1.01]. Lassi et al. [33] review six trials on pneumonia, and find an average risk reduction of 13% (RR = 0.87, 95% CI [0.81,0.94]). Yakoob et al. [9] re-analyzed existing randomized controlled trials in the areas and found a morbidity reduction of 19% (RR = 0.81; 95% CI [0.73, 0.90]).

#### Zinc and under-5 mortality

Brown et al. [13] found a relative risk of death of 0.94 [0.86-1.02]. In larger trials zinc supplementation reduced mortality of children 12 months old or older by 18% (RR = 0.82, 95% CI [0.70–0.96]), but had no effect on younger children. Yakoob et al. [9] found that Zinc supplementation alone was associated with a 9% reduction in all-cause mortality risk (RR = 0.91; 95% CI [0.82, 1.01]), but no effect in studies that also supplemented iron and folic acid. The point estimates were larger for mortality attributable to malaria and diarrhea, but not statistically significant.

### Modeling parameters

Based on the literature search results, the protective effect of zinc appeared relatively robust for diarrheal diseases, respiratory infections and all-cause mortality. There appears to be also growing evidence on a protective effect of zinc on malaria, while the evidence on diabetes, HIV and other diseases appears limited, so that the protective effect on these outcomes was assumed to be zero. While few studies focus on sub-group analysis, all reviewed studies suggested a noticeable heterogeneity in study outcomes related to diarrhea incidence. Study populations or subgroup analyses wherein the average child is stunted saw the largest protective effects of zinc interventions [8, 27, 34–37]. Conversely, studies or subgroups with an average length-for-age or height-for-age Z-score above −1.5 tended to see insignificant effects [37–44]. While there are two noteworthy exceptions to these patterns [45, 46] it appears plausible that zinc supplementation would primarily work among children with nutritional deficits. Zinc is known to inhibit growth, and stunting is widely considered the best anthropometric indicator of risk of zinc deficiency [47]. The proposition of increased morbidity protection amongst stunted children is analogous to the documented evidence that zinc supplementation elicits stronger growth responses amongst stunted children over 6 months of age [48]. In addition to baseline nutrition, age seems to be an important modifier of the health benefits generated by zinc, with children over 12 months of age benefitting more than infants where benefits appear limited [49].

For several of the parameters, point estimates varied considerably across studies. To capture this, three different scenarios were considered: a baseline scenario, an optimistic scenario, and a pessimistic scenario. The baseline estimates were chosen with the following decision rule: For each age/height-for-age/disease subgroup in our model, we chose the point estimate of the meta-analysis that most demographically matched our desired subgroup. If two meta-analyses covered the same demographic composition, we chose estimate from the more comprehensive meta-analysis. The “optimistic” scenario captures the highest effect reported in recent studies; the “pessimistic” scenario captures the lowest meta-estimate found in the reviews.

Health effect parameters were modeled to be equal across zinc delivery modes. Virtually all existing evidence on the health effects of preventive zinc are based on zinc supplementation trials, and we assume that the health benefits documented for supplementation trials can be achieved by all other delivery modes considered. This might not be the case if zinc is differentially absorbed across modes. In practice, absorption may be difficult in the presence of phytate, which is a known inhibitor of zinc absorption found in grains [50]. Iron can also act as an inhibitor of zinc absorption, though in lower doses [51] or when incorporated into meals [50–52] this effect is greatly dampened. More generally, the potentially deleterious interactions between simultaneous zinc and iron delivery should be carefully considered when delivery modes are developed [53–56].

Evidence for other modes is very limited A recent meta-analysis on micronutrient powders (MNP) including zinc neither finds no effect of MNP on zinc deficiency nor or child health [57]. A recent systematic review on food (formula, milk or porridge) fortification interventions suggests a positive impact of these interventions on serum zinc concentration, positive weight gains for zinc-deficient school age children, and positive height gains for infants with very low birth weight, but also highlights the “[.] *dearth of evidence for the impact of fortification strategies on morbidity and mortality outcomes in women and children*” [58]. For aqueously dissolved zinc, high rates of absorption seem feasible. Tran et al. [59] found that the fractional absorption of zinc ranged from 0.62 to 0.73 for aqueous zinc sulfate doses of 2 mg to 15 mg, with declining fractional absorption for 20 and 30 mg dosing. Solomons et al. [60] compare the absorption of aqueous zinc sulfate with the absorption of NutriSet tablets, and find that aqueous zinc administration doubles the bioavailability of zinc compared to the NutriSet tabs. To date, no evidence on the health impact of aqueous zinc is available.

In addition to the lacking evidence for the health benefits for delivery modes other than direct supplementation, evidence is also very scarce on the health benefits of supplemental zinc for children over 5, not allowing any general conclusions. Given this, the health benefits were assumed to be zero over the age of 5 under all scenarios. Table 1 shows the full set of parameter choices made under each scenario, separately for infants (age 0–11 months) and children (ages 12–59 months), and by stunting classification.

**Table 1:** **Health impact summary for infants and children of age 1**–**5**, **by stunting status**

	Baseline		Optimistic scenario		Pessimistic scenario	
**Health impact for stunted children ages 1-** **5**						
Risk reduction: Diarrhea	0.87	Yakoob [9]	0.73	Brown [13]	0.91	Patel [32]
Risk reduction: ALRI	0.86	Roth [28]	0.81	Yakoob [9]	0.94	Roth [28]
Risk reduction: Child mortality	0.82	Brown [13]	0.82	Brown [13]	0.94	Brown [13]
**Health impact for non-** **stunted children ages 1-** **5**						
Risk reduction: Diarrhea	1	Brown [13]	0.73	Brown [13]	1	Brown [13]
Risk reduction: ALRI	0.86	Roth [23]	0.81	Yakoob [27]	1	Lower limit
Risk reduction: Child mortality	0.82	Brown [8]	0.82	Brown [8]	0.94	Brown [8]
**Health impact for stunted infants** **(Age 0–** **11 months)**						
Risk reduction: Diarrhea	1	Brown [13] & Gulani [6]	1	Brown [13]	1	Brown [13] & Gulani [6])
Risk reduction: ALRI	1	Gulani [6]	0.81	Yakoob [9]	1	Lower limit
Risk reduction: Child mortality	1	Brown [13]	0.91	Yakoob & Brown [9, 13]	1	Brown [13]
**Health impact for non-** **stunted infants** **(Age 0–** **11 months)**						
Risk reduction: Diarrhea	1	Brown [13] & Gulani [6]	1	Brown [13]	1	Brown [13] & Gulani [6]
Risk reduction: ALRI	1	Gulani [6]	0.81	Yakoob [9]	1	Lower limit
Risk reduction: Child mortality	1	Brown [13]	0.91	Yakoob [9] & Brown [13]	1	Brown [13]

#### Household structure and baseline burden of diseases

Table 2 shows the assumptions made regarding household structure and haseline burden of disease. Average household size in the pooled least developed country group was assumed to be five in line with Bongaarts’ estimates [61]. Based on the World Population prospect data, each 100 households were assumed to host 15 infants, 59 children of ages 1–5, 127 5–14 year olds, and 299 individuals of age 15 or older [24]. Annual morbidity incidence was estimated at 3.42 cases of diarrhea, and 2.51 cases of ALRI per child and year using the DHS data [25]. Under-five mortality was estimated at 63 deaths per thousand live births in 2010, out of which 44 deaths occur in the first year of life. Assuming a uniform mortality distribution between ages 1 and 5 for simplicity, this converted to an annual mortality rate of 4.75 deaths per 1000 between ages 1 and 5. Thirty-two percent of children were estimated as being stunted [62]. Using Black’s estimates [63], mortality rates were allocated into mortality among stunted and non-stunted children, with 60 percent higher mortality odds in the stunting group.

**Table 2:** **Population and baseline health parameters**

Household structure		Source
Infant per 100 households	14.7	UN [24]
Children age 1–4 per 100 households: all	58.9	UN [24]
Children age 1–4 : stunted	18.8	UN [24], Black [63]
Children age 1–4: not stunted	40.1	UN [24], Black [63]
Children age 5–14 per 100 households	127.0	UN [24]
Persons ages 15 and older per 100 households	299.4	UN [24]
**Baseline burden of disease**		
Diarrhea	3.42	DHS [25]
ALRI	2.51	DHS [25]
Infant mortality: deaths per 1000	44	UNICEF [26]
Child mortality all: deaths per 1000	19	UNICEF [26]
Annual child mortality: stunted	6.4	UNICEF [26], Black [63], author’s calculations
Annual child mortality: not-stunted	4.0	UNICEF [26], Black [63], author’s calculations

#### Delivery cost

Several studies reviewed reported the cost for therapeutic treatments with zinc, which were used to estimate supplementation costs. While treatment doses are generally higher for therapeutic purposes than for supplementation (20 mg/day vs. 10 mg/day for supplementation), the average wholesale price is unlikely to differ much. Abdullah Brooks [64], Robberstad [29], and Srinivasan [65] suggest a price per 20 mg pill of between 2 – 6 cents, while Gitanjali [66] reports that a two week course can cost the Tamil Nadu government less than two rupees, equivalent to under a third of a penny per pill. We take the midpoint of 2–6 and assume 4 cents per pill in our baseline scenario, and use 6 cents (the highest estimate) and 1 cent (near the Gitanjali estimate) as pessimistic and optimistic scenarios (Table 3).

**Table 3:** **Estimated cost by delivery mechanisms**

Cost of zinc supplementation	Base case	Optimistic bound	Pessimistic bound	
Prophylactic supplements per dose	0.04	0.01	0.06	Brooks [64]
Annual per-person cost	14.60	3.65	21.90	Robberstad [29],
				Srinivasan [65],
				Gitanjali [66]
Micronutrient biscuits or sprinkles	0.08	0.03	0.08	Nga et al. [30],
Annual per-person cost	29.20	10.95	29.20	WFP
Water filter system per household	25	15	35	Vestergaard Frandsen
Water filter: zinc supplementation only per household	5	2	10	Vestergaard Frandsen

As to the distribution cost of micro-nutrients, the current estimate is about $ 0.08 per child and day [30], which we use both for our baseline and pessimistic calculation. Similar doses of zinc sulfate could also be added to children’s food through micronutrient powder or “sprinkles”, with an estimated cost of $ 0.03 or less according to the *World Food Programme* [67]. We take $0.03 as the optimistic scenario for micronutrient distribution.

The supplementation costs above were calculated as the cost to provide one treatment to one person on one day. Multiplying by 365 the daily costs in Table 3 and further multiplying by the number of treated children in 100 synthetic households from Table 1 yielded the total annual costs of these interventions. It is noteworthy that only Robberstad et al. [29] explicitly cost all the individual components of treatment delivery in the private market and aggregate them into a total. Gitanjali & Weerasuriya [66] and the WFP (the lowest estimates) specify they refer only to the price of zinc. The other estimates simply report per-treatment “cost” without specifying which components of the intervention are being considered.

Water filtration systems are currently under development, and would, according to the latest cost estimates provided by Vestergaard Frandsen cost about US$ 25–35 per year. This reflects an additional cost of about US$ 5 compared to standard water filtration systems for the zinc dispensing feature (optimistic US$ 2, pessimistic US$ 10). Table 3 provides a summary of the cost assumptions made for the cost effectiveness calculations.

#### Cost effectiveness results

Combining all health outcomes and impact estimates, systematic zinc supplementation among children under the age of five was estimated to annually avert 1.4. DALYs per 100 households in least developed countries. Using the most pessimistic and most optimistic health impact scenarios as upper and lower bounds for this estimate, a range of 0.5 - 3.1 DALYs per 100 households and year was found. Given that both the cost and impact estimates varied substantially, separate cost-effectiveness calculations were conducted under more or less optimistic cost and health impact scenarios. In Table 4, the main health impact estimate of 1.4 DALYs per 100 households was used in combination with a range of cost estimates. Column 1 shows the results for the distribution of zinc supplements to children under five. With an estimated baseline cost of 4 cents per pill and day (US$ 14.6 per year and child), the estimated cost per DALY is US$ 606; depending on the cost assumptions made, this number fluctuated between US$ 151 and US$ 908.

**Table 4:** **Incremental cost**-**effectiveness under the main health scenario**

Scenario	Cost per DALY: Prophylactic supplements for children 1–4 only	Cost per DALY: Biscuits	Cost per DALY Water filter systems	Cost per DALY water filter zinc addition only
Baseline cost	605.50	1211.00	878.66	175.73
Optimistic cost	151.38	454.13	527.20	70.29
Pessimistic cost	908.25	1211.00	1230.12	351.46

**Figure 1:**
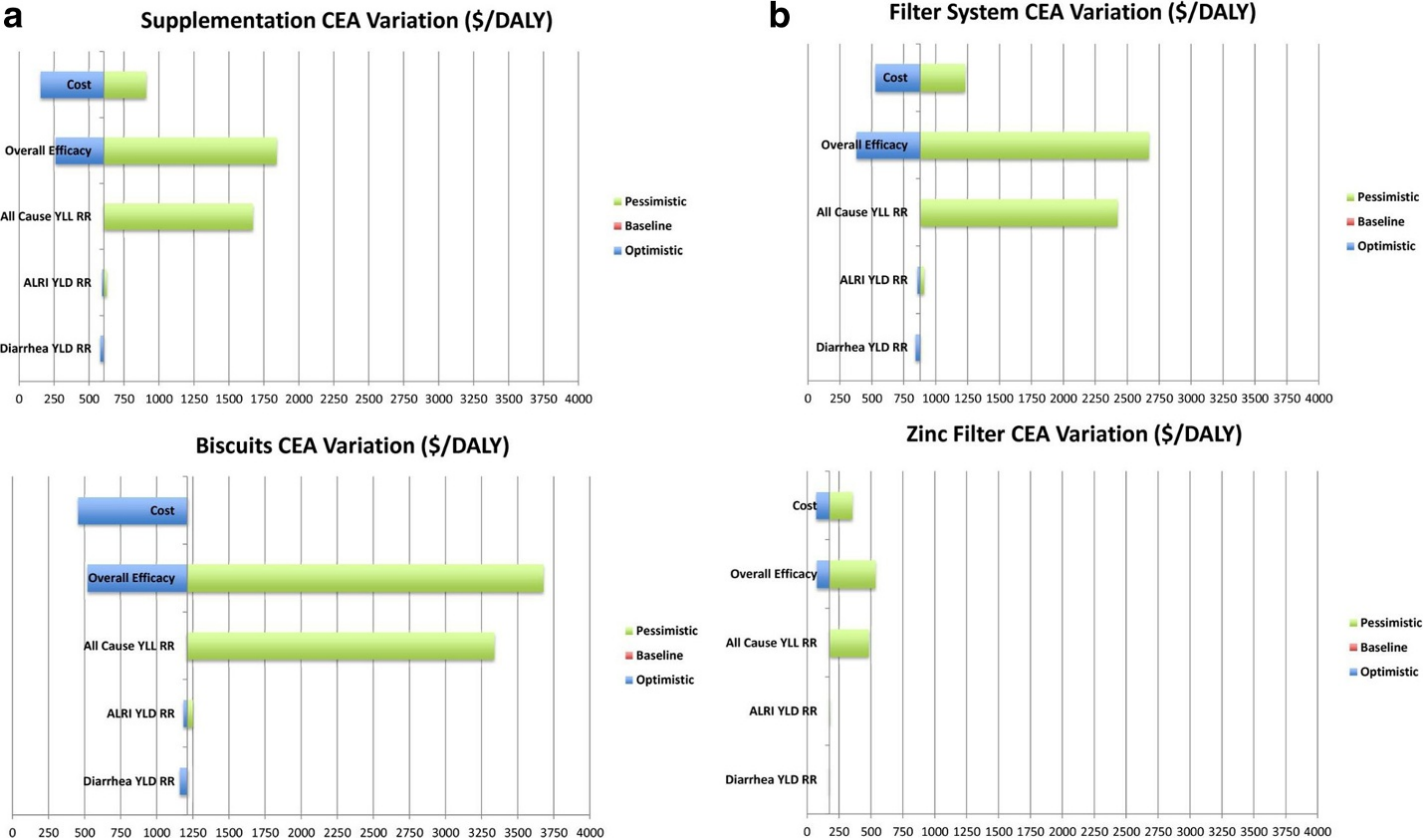
**Sensitivity analysis. a**: Tornado Chart for Supplementation and Biscuits. **b**: Tornado Chart for Water System and Water Filter.

Column 2 of Table 4 shows the results for micronutrient biscuits or the addition of micronutrient powder. Given the higher price per dose, the overall cost-effectiveness estimates looked slightly less favorable, with a main estimate of US$ 1211 per DALY, and optimistic and pessimistic bounds of US$ 454 and US$ 1211, respectively. Columns 3 and 4 of Table 5 show the results for water filter based systems. The cost structure of water filtration systems is fairly different from the distribution of supplements or biscuits, with the bulk of the attributable to manufacturing and only minor costs accruing once the filters are installed. Water filtration systems were assumed to last for two years on average, which translates to a cost of US$ 12.5 per household. With an average of 59 children in the critical age bracket, this translates to a cost of US$ 879 per DALY saved. Depending on the actual price of the water filtration system, this cost-effectiveness estimate ranges between US$ 527 and US$ 1230. When only the additional cost of adding zinc dispensing function to the water filtration systems was taken into account, the cost-effectiveness numbers looked substantially more favorable, ranging between US$ 70 and US$ 351 with a baseline estimate of US$ 175. In order to highlight the sensitivity of these estimates with respect to the underlying health impact, separate cost-effectiveness numbers were calculated for the four delivery mechanisms under the optimistic and pessimistic case health scenarios. The results of this sensitivity analysis are displayed in Table 5. On average, the cost per DALY drops to about 45% under the most optimistic health impact scenario, while it increases by about 200% if the most conservative health impact estimates are applied. Figure 1 summarizes the overall sensitivity of the results with respect to efficacy and cost.

**Table 5:** **Sensitivity analysis**: **health impact scenarios**

Health impact: Optimistic				
	Cost per DALY: Prophylactic supplements for children 1–4 only	Cost per DALY: biscuits	Cost per DALY water filter systems	Cost per DALY water filter zinc addition only
Baseline cost	260.68	521.36	378.28	75.66
Optimistic cost	65.17	195.51	226.97	30.26
Pessimistic cost	391.02	521.36	529.59	151.31
**Health impact:** **Pessimistic**				
	Cost per DALY: Prophylactic supplements for children 1–4 only	Cost per DALY: biscuits	Cost per DALY water filter systems	Cost per DALY water filter zinc addition only
Baseline cost	1838.45	3676.90	2667.82	533.56
Optimistic cost	459.61	1378.84	1600.69	213.43
Pessimistic cost	2757.67	3676.90	3734.95	1067.13

## Discussion

The results of this paper suggest that several interventions to address zinc deficiency are likely to be cost-effective under standard cost-effectiveness assumptions. For all delivery mechanisms analyzed, our preferred model specification suggests average costs per DALY strictly below US$ 1500, which implies that all interventions should be considered highly cost-effective in most countries currently classified as least developed countries under the WHO cost-effectiveness guidelines [22].

Several modeling assumptions limit the generalizability of the results, and are worth highlighting. First, all of our the cost-effectiveness calculations are based on the evidence from trials administering zinc in the form of tablets or syrup. Zinc absorption and the resulting health impact may differ across delivery modes, which would clearly affect the cost-effectiveness results presented in this paper. Second, while a large number of studies have analyzed the health benefits of zinc for children under 5, very little evidence is available for older children and adults. In the absence of better evidence, we assumed the health benefits to be zero outside of this age range. In the (rather likely) case that older populations also benefit from supplementation, the cost-effectiveness numbers reported here would clearly underestimate the true effectiveness of zinc interventions in general, and of water filtration systems in particular, where older household members are naturally exposed to zinc due to the household-based nature of the intervention. Third our model is calibrated to a representative household in a developing country. To the extent that a specific country has higher dependency ratios or higher rates of stunting, the average numbers used in this analysis will be an underestimate of the health impact achievable and the actual cost-effectivenessof the respective programs. We also assumed that adherence to each treatment was perfect, which is unlikely to hold in general, and with pill supplementation in particular. While both zinc supplementation through pills and multi-nutrient biscuits have been successfully implemented in controlled trials, limited evidence is available to date regarding the scalability and adherence to such interventions over time. Last, we only considered technologies currently available; new technologies to deliver zinc such as genetically modified crops are likely to improve the cost-effectiveness numbers substantially.

A potential shortcoming of our paper is that some evidence suggests our model may underestimate its efficacy. There is suggestive evidence that zinc supplementation can potentially reduce the burden of several other illnesses, including malaria, HIV, diabetes, depression. However, we found the empirical evidence too limited to include these impacts in the present study. For relevant discussions, see Yakoob et al. [9] Brown et al. [13], Zeng and Zhang [10], Sigfried [68], Humphreys [69], Islam and Loots [70],. Lai et al. [71], Nagala et al. [72], Kulier et al. [73], and Worthington et al. [74].

Overall, the health benefits achievable through systematic zinc supplementation appear large, while several important questions remain open with respect to its feasibility. Even if existing health system resources such as community health worker programs could be used to distribute zinc supplements on a regular basis, comprehensive zinc coverage through the distribution of zinc supplements would be a major challenge, and assuring adherence to treatment over time would undoubtedly be difficult. From a purely coverage-focused perspective, genetically modified crops would clearly be the first best option, since they would allow eliminating zinc deficiencies without requiring major behavioral changes from children and their parents. In the absence of such crops, which would likely take several years before being adopted by the agricultural sector even once developed, home-based water filtration systems could become an interesting alternative. Home based water filtration have been shown to be highly effective in reducing diarrhea [75] and thus constitute an effective intervention themselves in many developing country settings where water contamination at the point of use is common [75, 76]. At a price of US$ 20–30, these filters are likely to be highly cost-effective, but also likely too expensive to be currently affordable for the average household in low income countries [77]. To the extent that governments or international agencies are willing to support the mass distribution of household water filtration systems, moving towards filters with additional zinc features may become an interesting alternative. However, further field trials will be needed to assess the reliability of such systems as well as zinc absorption rates among individuals heterogeneous in their age, water consumption and zinc needs.

## Conclusions

The results presented in this paper suggest that preventive zinc supplementation to children of ages 1–4 should be considered a highly cost-effective intervention. Several delivery mechanisms promise to be cost-effective in principal. However, further research will be needed to establish the feasibility and impact of each mechanism at larger scale.

## Abbreviations

WHO: World Health Organization
DALY: Disability adjusted life year
ALRI: Acute lower respiratory illness
DHS: Demographic and Health Surveys
RR: Relative risk.
